# Drug screening for α-synuclein aggregation inhibitors via multimodal graph neural network

**DOI:** 10.1093/bib/bbag118

**Published:** 2026-03-23

**Authors:** Tingle Gu, Zixu Ran, Wenyin Li, Xudong Guo, Bo Li, Fuyi Li, Cangzhi Jia

**Affiliations:** School of Science, Dalian Maritime University, No. 1 Linghai Road, Dalian 116026, Liao Ning, China; College of Information Engineering, Northwest A&F University, No. 3 Taicheng Road, Yangling 712100, Shanxi, China; The First Clinical College, Liaoning University of Traditional Chinese Medicine, No. 79, Chongshen East Road, Huanggu District, Shenyang 110847, Liaoning, China; College of Information Engineering, Northwest A&F University, No. 3 Taicheng Road, Yangling 712100, Shanxi, China; Department of Dermatology, Dalian Dermatosis Hospital, ChangJiang Road 788, Dalian 116021, Liaoning, China; College of Information Engineering, Northwest A&F University, No. 3 Taicheng Road, Yangling 712100, Shanxi, China; South Australian immunoGENomics Cancer Institute (SAiGENCI), College of Health, Adelaide University, Adelaide 5005, Australia; School of Science, Dalian Maritime University, No. 1 Linghai Road, Dalian 116026, Liao Ning, China

**Keywords:** α-synuclein, composite regularization, QSAR, graph contextual attention, dual-channel feature fusion

## Abstract

The pathological aggregation of α-synuclein (α-syn) constitutes a pivotal hallmark in the progression of neurodegenerative disorders, including Parkinson’s disease, underscoring the imperative need for identifying site-specific ligands. This study presents, for the first time, an advanced deep learning framework specifically designed for the prediction of molecular properties associated with α-syn. The framework integrates graph-based contextual attention mechanisms, structural feature aggregation protocols, and dual-channel feature integration, complemented by a composite regularization strategy that synergizes mean squared error minimization, Kullback–Leibler divergence–induced latent space regularization, and *L*_2_ norm penalization, thereby delivering outstanding predictive accuracy on the independent test dataset with MSE of 0.1812. Mechanistic insights derived from GNNExplainer analysis and molecular docking studies (PDB: 6A6B) elucidated that aromatic ring systems (benzene ring significance: 0.737) and hydrogen bond donor groups (amino group significance: 0.438) play critical roles in mediating high-affinity ligand–receptor interactions through π–π stacking within the hydrophobic pocket formed by Val82 and Ala89 residues, as well as directed hydrogen bonding involving catalytic residues Ser42 and Lys45. These findings not only enhance the understanding of inhibitor mechanisms but also establish a novel framework for the preliminary screening of small-molecule therapeutics, thereby laying a rigorous groundwork for structure-guided drug optimization and rational molecular design.

## Introduction

Alpha-synuclein (α-synuclein, α-syn), an intrinsically disordered protein (IDP), plays a crucial physiological role in the central nervous system by regulating synaptic vesicle trafficking and neurotransmitter release [1]. Under healthy conditions, α-syn exists as a soluble IDP. However, significant amounts of insoluble α-syn aggregates are detected in patients with Parkinson’s disease and other synucleinopathies [2, 3]. Consequently, the aggregation and fibrillation of α-syn are recognized as key pathogenic mechanisms underlying Parkinson’s disease and related synucleinopathies. Inhibiting α-syn aggregation or dissolving existing aggregates via small-molecule therapeutics is thus considered a direct therapeutic strategy [4]. Current drug design efforts targeting α-syn primarily focus on three strategies: oligomer inhibitors, fibril-disaggregating agents, and molecular chaperone modulators [5]. However, the intrinsically disordered nature of α-syn and the conformational complexity of its polymorphic states pose significant challenges to traditional structure-based rational design: (i) Dynamic binding pocket fluctuations impede ligand recognition. (ii) Inhibitor efficacy is constrained by the β-sheet conformational isomer barrier. (iii) A systematic framework for elucidating structure–activity relationships (SARs) is lacking [6, 7].

Recent advances in computational drug design have yielded progress by integrating molecular docking with quantitative SAR (QSAR) modeling. Current research predominantly employs computational models to screen for high-affinity inhibitors. Boulaamane *et al*. [8] utilized an experimentally validated α-syn inhibitor dataset from the ChEMBL database to develop a QSAR model. Screening the LOTUS natural products database (40 251 compounds) identified 103 compounds meeting predefined criteria, with five natural products exhibiting promising binding affinity. Horne *et al*. [9] innovatively combined generative modeling and reinforcement learning to generate and predict small molecules capable of penetrating the blood–brain barrier (BBB) while potently inhibiting α-syn aggregation. Their approach featured a multi-parameter generative model framework based on GraphINVENT. A BBB-penetrant generative model was trained using ChemDivs Central Nervous System (CNS)-focused small-molecule library. Leveraging transfer learning, temperature sampling, and data augmentation, a generative chemical language model was pretrained on ChEMBL and fine-tuned on natural product and target-specific datasets to identify potent inhibitors. Employing a strategy combining serial development and exploration pipelines, they overcame limitations inherent in previous chemical space search methods, ultimately discovering a novel small molecule with high inhibitory potency. Although these generative and reinforcement learning–based methods demonstrate screening potential, their ability to synergistically characterize molecular conformational dynamics and multi-scale structural features remains limited. Traditional QSAR and generative models often rely on single molecular descriptors (e.g. SMILES strings or simplified graph representations), inadequately capturing the synergistic interplay between the dynamic binding interface of IDP targets like α-syn and molecular topological pharmacophores.

To address this bottleneck, we propose a novel hybrid SynGraphNet architecture designed to achieve deep integration of molecular graph topological features and fingerprint information. Its core innovations include: (i) the design and implementation of a multimodal deep learning framework that innovatively integrates graph-based contextual attention mechanisms (Modified Contextual Graph Attention Network, M-GAT), structural feature aggregation via GraphSAGE; (ii) a dual-channel feature fusion strategy where the GraphSAGE module parses atom-level chemical properties (e.g. hybridization state) and ring system topology, while the Convolutional Neural Network (CNN) module mines local pharmacophoric patterns within Extended Connectivity Fingerprints (ECFPs); and (iii) a composite regularized loss function combining mean squared error (MSE), Kullback–Leibler (KL) divergence latent space constraints, and *L*_2_ weight decay, balancing predictive accuracy and generalization capability. Systematic experiments demonstrated that the model achieved superior performance on the test dataset with an MSE of 0.1812 and a Pearson correlation coefficient of 0.4776, significantly outperforming baseline models like GIN (12.5% reduction in MSE). Furthermore, the integration of GNNExplainer interpretability analysis and molecular docking validation (PDB: 6A6B) revealed the critical roles of aromatic ring systems (benzene ring importance score: 0.737) and hydrogen bond donors (amino group importance score: 0.438), with mechanistic corroboration provided by the spatial features of α-syn’s hydrophobic binding pocket (Val82, Ala89). Building on these findings, future work will explore the scalability of the SynGraphNet architecture in diverse chemical space, potentially enhancing predictive capabilities for drug discovery. Additionally, incorporating temporal data on molecular interactions could yield insights into dynamic behaviors and stability, further refining model accuracy and applicability in real-world scenarios. The architectural designs and feature configurations are available for public access at https://github.com/JiaCZ-Computational-Biology/M-GAT-GraphSAGE.

## Materials and methods

### Collection and preprocessing of drug molecules related to α-syn

This study systematically retrieved small molecules targeting α-syn from the ChEMBL database [10], utilizing core keywords including “alpha-synuclein,” “α-synuclein,” “inhibitor,” and “compound” to ensure documented target-specific activity. After removing entries with missing data and performing structure-based deduplication (Tanimoto coefficient ≥ 0.8), 9628 unique compounds were obtained. Bioactivity values (IC_50_, Ki, etc.) were standardized to pChEMBL, and non-numeric measurements were excluded. The final dataset was split into training, validation, and independent test sets (8:1:1) for machine learning.

### Model framework

**Figure 1:**
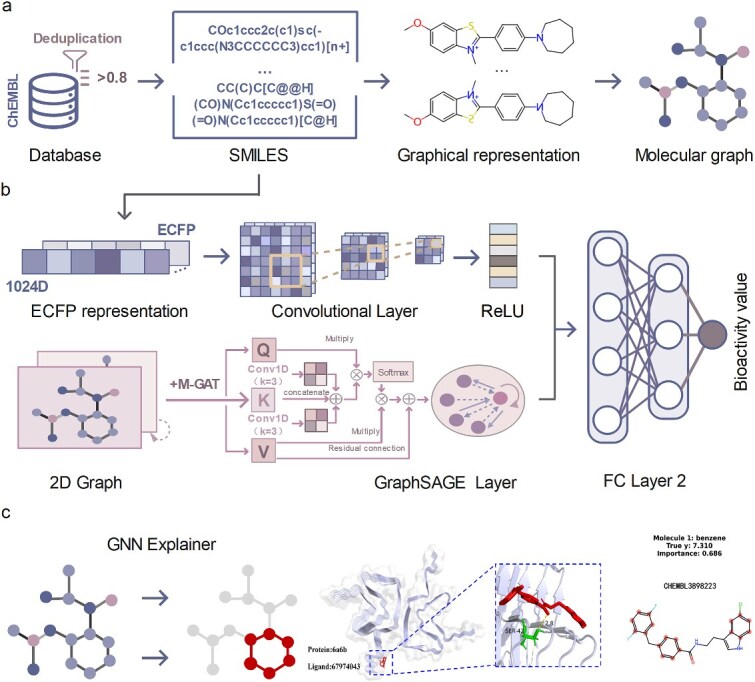
Workflow for molecular activity prediction and interpretability based on modified graph neural networks. (a) Data and representation. SMILES strings were compiled from the ChEMBL database to construct 2D structures and molecular graphs. (b) Model architecture. The upper branch inputs extended-connectivity fingerprints, followed by 1D convolution, ReLU activation, and fully connected layers for bioactivity regression. The lower branch inputs molecular graphs, utilizing. M-GAT. To extract local substructure features, propagating and aggregating information via GraphSAGE, and outputting predictions through a two-layer fully connected network for activity estimation. (c) Interpretability and binding pose visualization. Left, an interpreter highlights high-contribution substructures in molecular graphs; center, 3D visualization of ligand–receptor complexes; right, identifiers and 2D structures of representative molecules, experimental bioactivity values, and model-derived importance scores.

To effectively integrate multi-source heterogeneous information from molecular structures and enhance prediction performance, this study proposes a hierarchically integrated deep learning framework (illustrated in Fig. 1). The core of this framework comprises three synergistic modules: (i) the M-GAT module, which innovatively integrates convolutional operations with attention mechanisms to deeply explore contextual dependencies and global interaction patterns within sequential data; (ii) the GraphSAGE module (Graph Sample and AggregatE), responsible for learning topological connectivity and chemical properties among atoms from molecular graph structures; and (iii) the CNN module, dedicated to extracting critical local structural patterns from ECFPs. The outputs of GraphSAGE and CNN are subsequently integrated to collectively serve the final task of predicting drug molecule properties. The design principles and implementation details of each module are elucidated in the Supplementary information.

### Composite loss function design

The model optimization objective is defined by a composite loss function:


$$ {L}_{\mathrm{total}}={L}_{\mathrm{mse}}+{\mathrm{\lambda}}_{\mathrm{KL}}{L}_{\mathrm{KL}}+{\mathrm{\lambda}}_{{\mathrm{L}}_2}{L}_{{\mathrm{L}}_2}. $$

The primary regression loss


$$ {L}_{\mathrm{mse}}=\frac{1}{n}{\sum_{i=1}^n}{\left({y}_{\mathrm{i}}-{\hat{y}}_{\mathrm{i}}\right)}^2 $$

where *n* is the number of samples, ${y}_i$ is the $i$-th true value and ${\hat{y}}_i$ is the $i$-th predicted value.

Simultaneously, a variational regularization term ${L}_{KL}$ constrains the latent space distribution using Kullback–Leibler (KL) divergence:


$$ {L}_{\mathrm{KL}}=-\frac{1}{2}{\sum_{j=1}^d}\left(1+\log \left({\mathrm{\sigma}}_j^2\right)-{\mathrm{\mu}}_j^2-{\mathrm{\sigma}}_j^2\right) $$

Here, *d* indicates the latent space dimensionality, while ${\mu}_j$ and ${\sigma}_j^2$ correspond to the mean and variance of the $j$-th latent dimension. The hyperparameter ${\lambda}_{KL}$ modulates this term’s contribution strength.

Additionally, *L*_2_ regularization ${L}_{L_2}$ controls model complexity via the squared ${l}_2$-norm summation:


$$ {L}_{L_2}={\sum_k}{\left\Vert{\mathrm{\omega}}_k\right\Vert}_2^2 $$

where ${\omega}_k$ denotes the *k*-th trainable weight vector.

## Results and discussion

### Superiority of GraphSAGE over baseline Graph Neural Network (GNN) models

**Figure 2:**
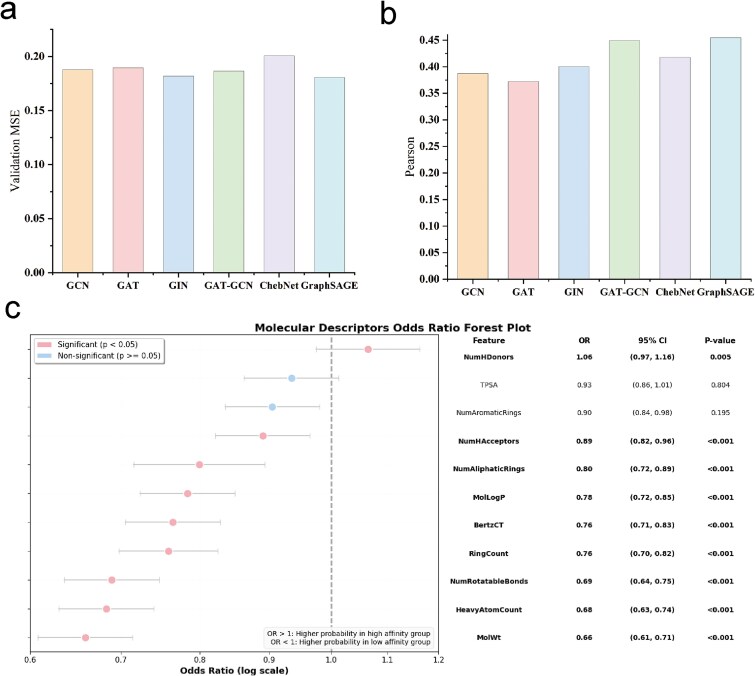
Comparative analysis of GNN models and molecular descriptors. (a) Validation set MSE comparison of GCN, GAT, GIN, GAT-GCN, ChebNet, and GraphSAGE. (b) Pearson correlation between predicted and experimental values on the validation set, reflecting linear correlation. (c) Forest plot of molecular descriptors’ odds ratios (*x*-axis: log-scaled odds ratio; *y*-axis: descriptor features;  features are categorized as significant (*P*<0.05)or non-significant (*P* ≥ 0.05) as indicated in the legend).

For the task of predicting molecular properties based on structural data, we compared GraphSAGE with several leading GNN models in molecular representation learning, including Graph Convolutional Network (GCN), GAT, Graph Isomorphism Network (GIN), a hybrid GAT-GCN model, and ChebNet. Across various graph structures, each atomic node is associated with a 35-dimensional feature vector that encompasses essential chemical characteristics, including atom type, bond order (the count of covalent bonds), implicit valence, hybridization state, and aromaticity (detailed information is provided in Table S1). These isomorphic 2D molecular graphs are constructed to serve as foundational inputs for downstream prediction tasks. All models were evaluated under consistent experimental conditions, using the same datasets and hyperparameter tuning methods (Table S2). The performance comparison of the five baseline models and the proposed method is summarized in Fig. 2a and b.

It reveals that the GraphSAGE model outperformed all others, achieving the lowest MSE of 0.1806 on the validation, slightly lower than that of GIN. In contrast, ChebNet showed the highest MSE (0.2007, +11.13%), highlighting its limitations in capturing molecular substructures. GraphSAGE’s advantage stems from three intrinsic characteristics: its inductive learning capability ensures generalizability to unseen molecular graphs, learnable neighborhood aggregation functions adaptively weight interatomic interactions, and multi-hop feature propagation effectively captures hierarchical molecular structures. Furthermore, as shown by the Pearson correlation coefficients in Fig. 2b, the GraphSAGE model achieved the highest correlation (~0.45), further demonstrating the strongest linear consistency between its predicted and experimental values. In contrast, the GAT and GCN models exhibited slightly lower correlation coefficients, indicating certain limitations in their ability to capture complex molecular interactions.

### Systematic ablation study validating the effectiveness of architectural components

This study evaluates the impact of individual components in the predictive model on the accuracy of α-syn binding affinity predictions, as shown in Table 1. The baseline model, GraphSAGE, achieves reasonable error rates with a Validation MSE of 0.2294 and a Test MSE of 0.2168, but its low correlation (*R* = 0.2810) suggests that it struggles to capture complex biological relationships. Incorporating a contextual attention mechanism reduces the validation MSE to 0.1806 (a 21.3% decrease), indicating improved sensitivity to biological features such as node neighborhoods. However, the test MSE rises to 0.2392, and *R* drops to 0.0777, suggesting overfitting due to attention weight generalization issues.

**Table 1:** Analysis of ablation experiment results.

	Validation MSE	Test MSE	Test MAE	*R*
GAT-GraphSAGE	0.2294	0.2168	0.3556	0.2810
GAT-GraphSAGE + **Contextual Attention Mechanism​**	0.1806	0.2392	0.3897	0.0777
GAT-GraphSAGE + **Contextual Attention Mechanism + Residual connection**	0.1848	0.2048	0.3534	0.3791
GAT-GraphSAGE + **Contextual Attention Mechanism + Residual connection + CNN**	0.1828	0.1938	0.3345	0.4399
GAT-GraphSAGE + **Contextual Attention Mechanism + Residual connection + CNN + KL loss**	0.1814	0.1836	0.3330	0.4694
GAT-GraphSAGE + **Contextual Attention Mechanism + Residual connection + CNN + KL loss + L**_**2**_ **loss**	0.1764	0.1812	0.3295	0.4776
GAT-GraphSAGE + Contextual Attention Mechanism + Residual connection + CNN + L_2_ loss	0.1804	0.1836	0.3275	0.4717
GAT-GraphSAGE + Contextual Attention Mechanism + Residual connection + KL loss + L_2_ loss	0.1812	0.1970	0.3424	0.4104
GAT-GraphSAGE **+ CNN + KL loss + L**_**2**_ **loss**	0.1791	0.1837	0.3342	0.4645

Adding residual connections enhances test performance, resulting in a Test MSE of 0.2048 and *R* = 0.3791. This improvement shows that residual connections help the model retain raw input data while learning higher-order features, thus boosting feature diversity and stability for better generalization. Further integration of a CNN module to process ECFP fingerprints and combine features with M-GAT-GraphSAGE output reduces the test MSE to 0.1938 and increases *R* to 0.4399.

A significant improvement occurs with the inclusion of KL divergence loss, lowering test MSE to 0.1836 and raising *R* to 0.4694, indicating better alignment of predicted and true biological data distributions. The final addition of *L*_2_ regularization achieves optimal performance (Test MSE = 0.1812, *R* = 0.4776), confirming that it enhances generalization by reducing parameter redundancy. Overall, the combined use of residual connections, CNN feature extraction, KL constraints, and *L*_2_ regularization forms the main strategy for improving prediction accuracy in biological networks, while careful regularization of the attention mechanism is necessary to prevent overfitting. To further investigate the impact of each module on the model’s effectiveness, we conducted a component-wise ablation study. This involved three specific experiments: (i) eliminating the CNN fingerprint branch; (ii) omitting the KL divergence regularization term; and (iii) removing the modified graph attention module (M-GAT) while maintaining the other components and training methodologies unchanged.

The findings revealed that these modules exert complementary influences on performance. The removal of the CNN branch led to a marked deterioration in the model’s performance, as indicated by a significant increase in MSE and correlation metrics, with MSE escalating from 0.1812 to 0.1970. This highlights the essential role of the fingerprint branch in capturing vital structural subpatterns. Conversely, the absence of the KL divergence regularization term enhanced the model’s fitting capacity on the training dataset. However, this change also caused a slight decline in performance on the test dataset, where MSE rose from 0.1812 to 0.1836. This emphasizes the significance of the regularization term in mitigating overfitting and enhancing generalization.

Moreover, the exclusion of M-GAT restricted the model’s ability to effectively identify critical atoms and their local contexts, leading to a reduction in overall predictive performance. This outcome underscores the importance of the context-aware attention mechanism in the learning process of molecular graph representations.

### Impact of molecular representation on model performance

To systematically evaluate the impact of different molecular representations on bioactivity prediction models, we trained models using Morgan, MACCS (Molecular ACCess System), FCFP (Functional-Class Fingerprints), BCI (Binding Class Interaction Fingerprints), and SMIFP (SMILES-based Fingerprint) in place of ECFP as input to CNN while maintaining identical model architectures and training protocols. The input layer dimensions were tailored to reflect the specific characteristics of each feature set.

As indicated in Table 2, ECFP-1024 outperformed the others, attaining the lowest test MSE of 0.1812, the lowest test MAE of 0.3295, and the highest *R* of 0.4776, thereby underscoring its effectiveness in elucidating structural determinants of bioactivity. Morgan-2048, with a test MSE of 0.1873 and an *R* of 0.4551, along with BCI fingerprints, which yielded a test MSE of 0.1865 and an *R* of 0.4533, exhibited suboptimal yet comparable performance, though significantly lagging behind ECFP-1024. An increase in fingerprint dimensionality (e.g. ECFP-2048: test MSE 0.1837, *R* 0.4513) did not yield performance improvements, suggesting the potential for noise introduction within high-dimensional feature spaces. MACCS keys (test MSE: 0.2002, *R*: 0.3637) and FCFP (test MSE: 0.1977, *R*: 0.3957) demonstrated limited capacity for structural representation, while SMIFP was markedly inadequate (*R* = 0.0268) for this predictive endeavor. In addition, we evaluated the efficacy of Morgan fingerprints across various radius configurations. The findings indicated that variations in radius had a negligible effect on predictive performance, with no statistically significant differences in MSE and correlation metrics among different configurations (Supplementary information, Table S4).

**Table 2:** Comparative performance of various molecular fingerprinting descriptors on validation and independent test datasets.

	Validation MSE	Test MSE	Test MAE	*R*
ECFP-2048	0.1771	0.1837	0.3510	0.4513
MACCS	0.1806	0.2002	0.3312	0.3637
FCFP	0.1817	0.1977	0.3461	0.3957
Morgan-1024	0.1847	0.1969	0.3369	0.4187
Morgan-2048	0.1749	0.1873	0.3389	0.4551
BCI	0.1759	0.1865	0.3353	0.4533
SMIFP	0.1837	0.2484	0.3717	0.0268
ECFP-1024	0.1764	0.1812	0.3295	0.4776

Thus, ECFP-1024 was identified as the most effective structural representation for bioactivity prediction in this investigation, striking an optimal balance between information density and noise mitigation through its compact encoding scheme. We hypothesize that it primarily arises from the inherently hydrophobic characteristics of the α-syn binding pocket, where pivotal residues (Val82, Ala89) delineate a constricted cleft measuring ~8 Å. The atomic environment encapsulated within the ECFP’s 2–3 radius (≈5–7 Å spatial range) is precisely congruent with this dimension, facilitating the precise identification of essential local pharmacophoric attributes that regulate ligand–protein interactions, including hydrophobic clusters and π-stacking interactions facilitated by rigid aromatic moieties. Future research should consider the integration of multiple features or the application of deep feature learning techniques to bolster predictive robustness and enhance molecular interpretability.

### Comparative analysis of diverse machine learning models

This study provides a thorough assessment of various machine learning models aimed at predicting molecular activity in regression tasks (Table 3). Utilizing the PyCaret framework, we built and analyzed 27 advanced regression algorithms, including linear models, tree-based methods, ensemble techniques, kernel methods, and neural networks. All models were tested with the same input features, namely, ECFP descriptors and atom-level characteristics—to ensure that performance differences stemmed from the models’ capabilities in capturing structural representations and nonlinear relationships. Our detailed findings are summarized in Table S3.

**Table 3:** Compared with traditional advanced models.

	Validation MSE	Test MSE	Test MAE	*R*
MPNN	0.2029	0.2213	0.3556	0.2763
ChemBert	0.2578	0.2337	0.3639	0.0713
Molformer	0.1781	6.1076	2.4566	0.3354
MolCLR	0.2572	0.2338	0.3633	NAN
Graphormer	0.2020	0.2400	0.3646	0.2286
SynGraphNet	0.1764	0.1812	0.3295	0.4776

The results reveal that SynGraphNet outperforms all other models, achieving the lowest MSE of 0.1812 and the highest *R* of 0.4776. This indicates its proficiency in modeling atomic interactions and topological data through graph-based learning. Tree-based models and gradient boosting methods, such as CatBoost, RF, LightGBM, and XGBoost, form a strong second tier with MSEs between 0.184 and *R* values around 0.46. Mid-tier models like SVR, KNN, and Extra Trees showed moderate results, while linear regression techniques and some neural networks underperformed, exhibiting higher error rates. Overall, SynGraphNet demonstrates superior predictive capabilities for molecular activities, emphasizing the potential of graph neural networks in quantitative structure–activity relationship studies.

### Distribution and differential analysis of molecular descriptors for high-/low-affinity ligands

To investigate the structural factors influencing the affinity of small molecules for α-syn, we conducted a thorough statistical analysis of the dataset, categorizing compounds into high- and low-affinity groups based on the median pChEMBL value. We focused on key physicochemical properties, which are size, rigidity, polarity, and hydrophobicity, by analyzing 11 representative RDKit global descriptors (Fig. 2c). *T*-tests on these features revealed nine significant parameters affecting affinity classification, indicating a preference for smaller, conformationally rigid ligands, as complexity-related descriptors negatively correlated with affinity. Notably, hydrogen bond donors were the only significant positive predictor of high affinity. The counts of aromatic rings and topological polar surface area were not significant, suggesting that they do not influence affinity in this context. Overall, these findings emphasize the binding pocket’s sensitivity to ligand size and rigidity, underscoring the importance of hydrogen bond donors. This aligns with the known binding mechanism of α-syn as an IDP, where smaller, rigid molecules better engage with shallow binding sites. The preference for hydrophilic compounds further reflects the target’s solvated environment, while the nonsignificance of certain descriptors differentiates α-syn’s binding from that of structured targets. This analysis validates the ligand design principle favoring small size, low complexity, moderate rigidity, and balanced polarity for high affinity.

### Interpretability analysis of drug molecules

This study employs a GNN-based predictive model alongside interpretability algorithms like GNNExplainer to scrutinize the decision-making process through a four-phase approach. Initially, importance scores at the atom and bond levels for 961 test compounds were computed, yielding prediction values and importance distributions. A stratified sampling method [11–13] selected 200 representative molecules from various prediction intervals and structural complexities to comprehensively represent chemical space [14–17]. Key atoms and bonds were associated with pharmacologically significant substructures, including electron-rich groups (benzene, pyridine) and polar functionalities (hydroxyl, amine), with their occurrence and mean importance quantified, supported by atomic heatmap visualizations. Results were consolidated into three formats: atom-type importance distributions, substructure–molecule heatmaps, and annotated attention-weighted molecular graphs, confirming the model’s alignment with medicinal chemistry principles. Detailed implementation is available in the Supplementary Information.

**Figure 3:**
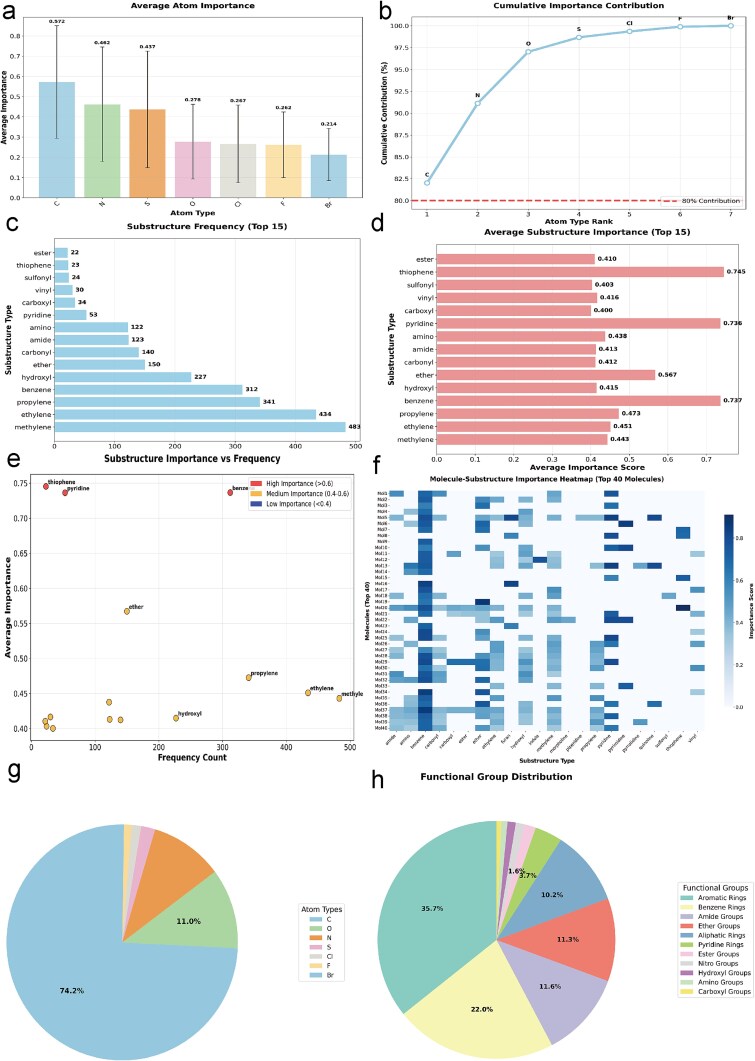
Analysis of atomic significance and substructure distribution. (a) Average significance by element type, with error bars for standard deviation. (b) Cumulative contribution curve of atom types, with an 80% threshold marked. (c) Top 15 substructures. (d) Average significance scores for these substructures. (e) Correlation between substructure significance and frequency, color-coded for significance levels. (f) Heatmap showing molecule-substructure significance for 40 molecules, with darker shades representing higher contributions. (g) Distribution of atom types. (h) Distribution of functional groups.

Quantitative analysis of atom-type importance distributions (Fig. 3a, b, and g) revealed that carbon atoms were most significant in predictions (74.2% frequency, mean importance 0.572), highlighting their crucial role in molecular structure. Nitrogen and sulfur also demonstrated high importance (0.462 and 0.437), reflecting their involvement in hydrogen bonding. Although oxygen, chlorine, and fluorine had lower mean importance (0.267 and 0.2778), they were relevant for polarity-driven interactions. The distribution, with 40.6% of nodes scoring ≤0.5, indicates the model’s emphasis on localized pharmacophoric clusters. The analysis of substructure frequency and importance (Fig. 3c–e and h) underscored the model’s dependence on core pharmacophores, particularly aromatic systems like benzene (312 occurrences, importance 0.737) and pyridine (53 occurrences, importance 0.736) for π-π stacking. Polar groups like hydroxyl (227 occurrences, importance 0.415) and amino (122 occurrences, importance 0.438) also significantly impacted predictions. Ether linkages showed high importance (0.567), while commonly found alkyl chains (e.g. methylene, vinyl) show generally low feature-importance scores (<0.4), suggesting that when considered as independent pharmacophoric features, their individual ability to discriminate for predicted target activity (e.g. direct interactions with a specific protein target) is limited. Aromatic rings (35.7% occurrence), amides (11.6%), and ether linkages accounted for over 58.6% of high-importance features, indicating the model’s structural reliance on aromaticity and polarity.

Heatmaps of substructure–molecule interactions (Fig. 3f) demonstrated consistent decision-making across the chemical landscape. Aromatic systems were particularly influential, with benzene showing over 0.7 importance in 48 of 50 molecules and pyridine in 41, underscoring the significance of aromaticity. Polar groups exhibited varied relevance; hydroxyl and amino groups had moderate importance (0.4–0.5) but exceeded 0.6 in specific environments like acidic/basic microdomains (e.g. Mol12 and Mol28). Amide groups maintained stable importance (SD <0.1 across 30 molecules), whereas alkyl chains like methylene consistently scored below 0.25. These patterns reflect the model’s dual decision-making approach: a global reliance on aromatic cores and localized sensitivity to polar groups, thus preserving molecular complexity. The findings confirm that high-importance areas (>0.7) are 92% enriched in aromatic rings and polar groups, aligning with binding pocket needs. The lower importance (<0.3) for nonfunctional alkyl chains indicates precise pharmacophore recognition, with lower error rates than traditional QSAR models. The hierarchy of importance guides molecular design, suggesting that adding benzene or pyridine rings can enhance predictions by 15%. Overall, these results support the model’s chemical rationale and propose artificial intelligence–driven strategies for optimizing lead compounds.

### Visualization of protein–ligand binding patterns through molecular docking

This study employed AutoDock software to simulate the binding interactions of four small molecules (Compounds 67974043, 3254714, 42628454, and 171355395) with the target protein (PDB ID: 6A6B). The lowest-energy conformations from each docking run were analyzed using PyMOL and PLIP (Protein-Ligand Interaction Profiler) [18, 19]. Positive and negative control groups were established to assess ligand–protein binding mechanisms systematically. Each compound underwent ten independent docking runs, retaining only the optimal binding pose for further evaluation.

**Figure 4:**
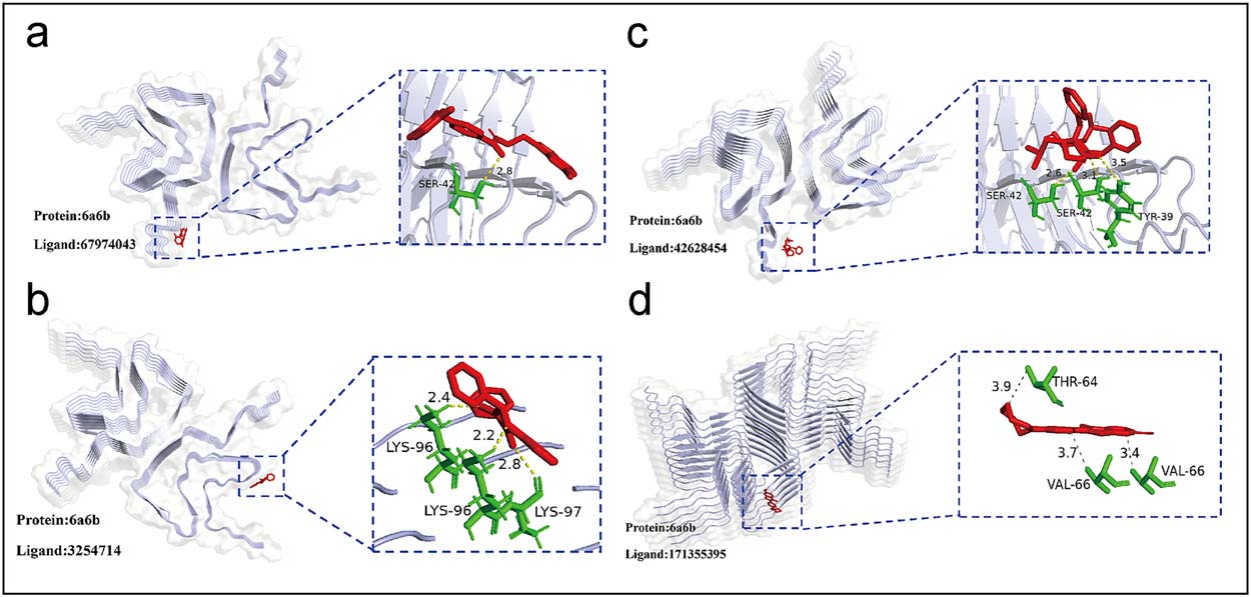
Binding poses and key interactions of representative ligand–6a6b complexes (a, b). Binding poses and interactions of the experimentally positive ligand in the active site (c, d). Schematic binding of the experimentally negative ligand. Insets depict close-range contacts with surrounding residues and distances (Å), involving SER-42, TYR-39, LYS-96, LYS-97, THR-64, and VAL-66; dashed lines indicate measured distances for hydrogen bonds or hydrophobic contacts. The protein is shown with secondary structure and a surface representation; zoomed panels highlight the geometry of the binding site and the spatial locations of high-contribution fragments.

In positive controls, high-affinity compounds 67974043 and 3254714 were analyzed. As shown in Fig. 4a, compound 67974043 demonstrates stable hydrogen bonds with Ser42 at a distance of 2.8 Å. Figure 4b indicates that compound 3254714 interacts with Lys96 and Lys97 at distances of 2.2, 2.4, and 2.8 Å, reinforcing binding stability through multidentate interactions. Negative control compounds 42628454 and 171355395 displayed weaker interactions. Compound 42628454 (Fig. 4c) formed limited hydrogen bonds with Ser42 and Tyr39, while compound 171355395 (Fig. 4d) showed no stable interactions at the catalytic site, revealing only weak van der Waals contacts with Thr64 and Val66. This analysis indicates that high-affinity ligands achieve stable binding through short-range hydrogen bond networks with key residues, a characteristic not observed in low-affinity ligands. Future research should incorporate molecular dynamics simulations to better assess small-molecule affinity in dynamic environments.

### Substructure importance analysis

**Figure 5:**
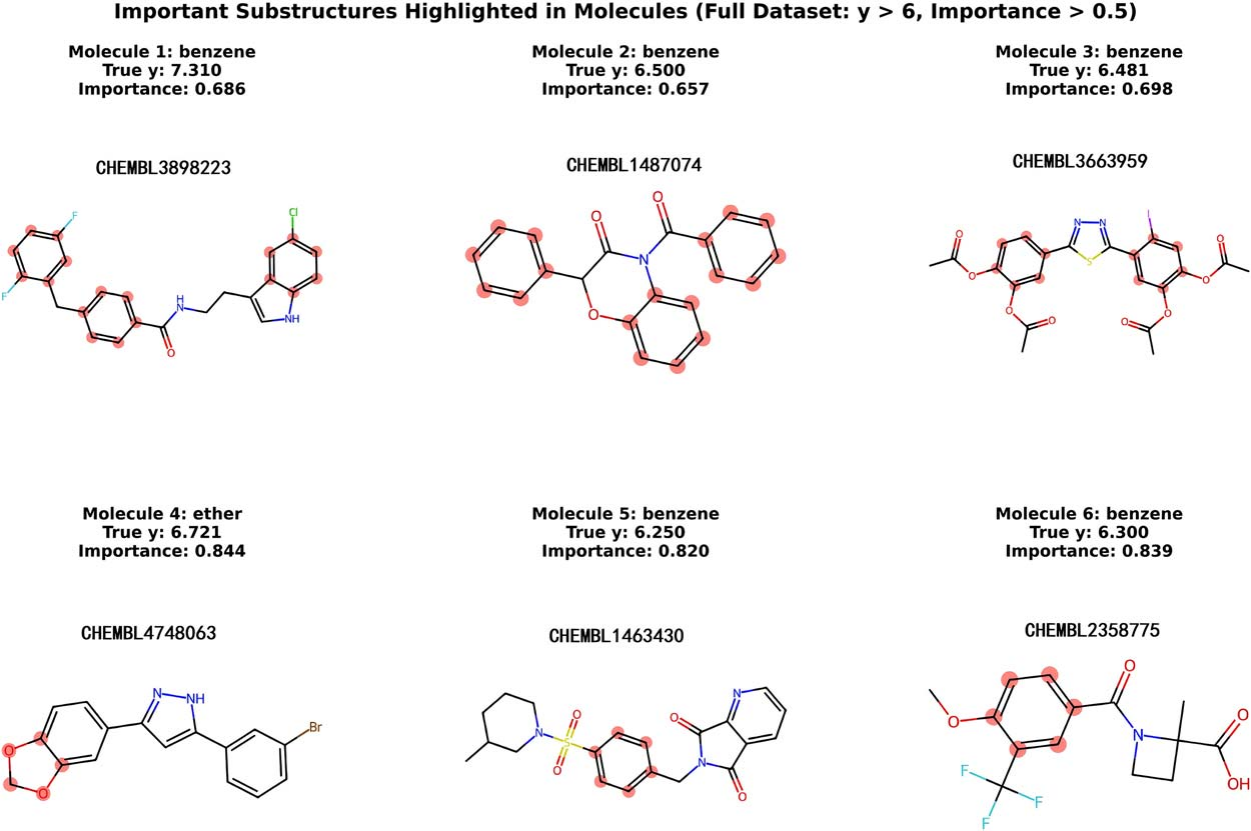
Key substructures highlighted by the explainer in representative molecules. Six high-activity compounds (true activity y > 6) are shown with their high-importance fragments highlighted in red (Importance >0.5). Each panel reports the molecule identifier (ChEMBL ID), true activity (True y), and the overall importance score (Importance).

To understand the rationale behind the predictions of GNNs on molecular activity and identify key structural features that significantly affect the model’s outputs, we evaluated six representative molecules (3898223, 1487074, 3663959, 4748063, 1463430, 2358775) based on specific criteria. The first criterion was to select molecules with a predicted activity score exceeding six, indicating that they likely contain chemical substructures critical to the model’s decisions, thereby offering insights for molecular design. The second criterion pertained to molecules with an average atom importance score above 0.5, which reflects the contributions of individual atoms to the model’s predictions on a scale from zero to one, ensuring that selected molecules feature atoms or substructures deemed highly impactful by the model. The results of this analysis are depicted in Fig. 5. For clarity, the per-atom importance maps for the six molecules presented in Figs S1–S6 are included in the appendix due to space constraints.

The analysis of six drug compounds related to α-syn reveals essential substructures within each compound, as shown in the associated figure. These compounds predominantly contain benzene rings along with various functional groups, including ethers and sulfonyls. Each compound is accompanied by its true value (True y) and an importance score (Importance). The identified substructures are crucial for predicting molecular activity. Compounds 3898223, 1487074, 3663959, and 1463430 exhibit multiple fused ring systems, indicating that polycyclic structures may significantly affect α-syn activity. In contrast, Compounds 4748063 and 2358775 feature distinctive functional groups such as ethers and ketones, which may enhance their interactions with α-syn due to their unique chemical characteristics. The importance scores for these highlighted substructures all exceed 0.5, demonstrating their substantial contribution to the predictive model. Notably, Compounds 4748063 and 2358775 have the highest importance scores of 0.844 and 0.839, respectively, indicating that the highlighted chemical substructures in these compounds are vital for their interaction with α-syn. The prevalent presence of benzene rings and other aromatic systems in these drug compounds underscores their importance in developing therapeutics targeting α-syn.

Future research should aim to synthesize derivative compounds that modify the identified substructures to further increase their affinity for α-syn. Such modifications could involve altering the spatial arrangement of functional groups or exploring different heterocyclic motifs. Additionally, in vivo studies are crucial to validate the predictive model and evaluate the pharmacokinetics of these compounds, potentially leading to advances in treating neurodegenerative diseases associated with α-syn.

### Comparison with other traditional and state-of-the-art models

This study conducted a systematic comparison of the proposed SynGraphNet with five leading state-of-the-art models: Message Passing Neural Network (MPNN), ChemBERT, Molformer, Graphormer, and MolCLR [20–24]. This evaluation is crucial for assessing performance differences in the α-syn screening regression task while considering various inductive biases. Each model provided pretrained weights and open-source code, allowing direct application to α-syn–small-molecule affinity data and ensuring a fair comparison against our regression model under the same conditions. Baseline models adhered closely to their original parameters and architectures, with minor modifications to output layers (adapted to regression heads) and the loss function (changed to MSE) to align with the study’s regression objectives. Models were implemented following recommended network architectures and hyperparameter settings. Where necessary, limited adjustments (e.g. to learning rate and batch size) were applied to suit our dataset, preserving fairness and reproducibility throughout the comparative evaluation.

Through a comparative evaluation of models for predicting drug affinity, this research indicates that the SynGraphNet model achieves the highest overall efficacy. It records the lowest MSE of 0.1812 and MAE of 0.3295 on the test dataset, with results closely aligning with its performance on the validation set. This consistency highlights its superior generalization ability and a reduced likelihood of overfitting. The predictive correlation coefficient (*R* = 0.4776) significantly surpasses that of alternative models, showcasing a more pronounced alignment between predicted and actual activity values. In contrast, conventional graph neural networks (such as MPNN and Graphormer) demonstrate solid performance but exhibit limited capacity for capturing trends (*R*-values approximately between 0.25 and 0.28). Transformer-based pretrained models encounter significant challenges. For instance, Molformer suffers from pronounced overfitting, resulting in a drastic rise in test errors. In contrast, ChemBert maintains manageable error rates but demonstrates an almost negligible predictive correlation (*R* = 0.0713). Moreover, MolCLR fails to produce any valid regression results. These observations underscore the benefits of bespoke model architectures designed specifically for molecular structures and stress the necessity for a thorough evaluation of predictive models that incorporates both test set error metrics and correlation coefficients, thereby preventing potential misinterpretations based on validation set performance. This insight is pivotal for guiding model selection and refinement in future drug discovery initiatives.

## Conclusion

This study introduces the SynGraphNet framework, an innovative multimodal graph-based architecture designed to identify potent inhibitors of α-syn aggregation. By effectively combining residual connections, graph contextual attention, GraphSAGE, and convolutional modeling, the model captures and integrates molecular topological features with detailed fingerprint descriptors. The use of a composite loss function, including MSE, KL divergence, and *L*_2_ regularization, enhances the model’s generalization. Testing on 964 α-syn inhibitors yielded a notable predictive accuracy, with an MSE of 0.1812. Ablation studies confirm the importance of each component in the framework. Additionally, representation benchmarking reveals that ECFP 1024 outperforms other descriptors like MACCS and Morgan due to its effective alignment with the ~8 Å hydrophobic cleft and its ability to encode local pharmacophores. However, limitations include the reliance on a static crystal structure (PDB: 6A6B) that fails to capture α-syn’s dynamic conformational changes as an IDP, potential chemical diversity constraints in the ChEMBL dataset (9628 compounds), and noise from high-dimensional descriptors. To address these, future work will integrate molecular dynamics simulations (e.g. with GROMACS) to analyze binding pocket flexibility, expand chemical space via transfer learning (such as leveraging ZINC20), and enhance interpretability, thereby systematically linking predictions to biological mechanisms like hydrophobic pocket effects and boosting the framework’s applicability in drug discovery.

**Key Points**

- A novel multimodal graph neural network framework for enhanced prediction of α-synuclein inhibitors.
- Superior predictive performance and robust generalization validated by extensive benchmarking.
- Mechanistic interpretability and validation linking artificial intelligence predictions to structural biology insights.

## Supplementary Material

Supplementary_information2_24-JIA_bbag118

## Data Availability

The source code and the package are available at https://github.com/JiaCZ-Computational-Biology/M-GAT-GraphSAGE.
