# Supplementary material for: Drug screening for α-synuclein aggregation inhibitors via multimodal graph neural network
Source: Brief Bioinform. 2026 Mar 23;27(2):bbag118. doi: 10.1093/bib/bbag118 (PMC13006971; doi:10.1093/bib/bbag118)
Supplement: Supplementary_information2_24-JIA_bbag118 [file supplementary_information2_24-jia_bbag118.docx]

**Supplemental Information**

**Table of Contents**

[**1. Materials and methods** 2](#_Toc7697)

[1.1 Collection and preprocessing of drug molecules related to α-syn 2](#_Toc32525)

[1.2 Convolutional neural networks module 2](#_Toc9571)

[1.3 The modified contextual graph attention network module (M-GAT) 3](#_Toc948)

[1.4 Graph sample and aggregate module (GraphSAGE) 4](#_Toc11575)

[1.5 Training normalization and evaluation denormalization 4](#_Toc31228)

[1.6 Evaluation metrics 5](#_Toc13235)

[1.7 Experimental setup 5](#_Toc29358)

[**2. Supplementaey tables** 6](#_Toc19660)

[**3. Supplementary figures** 8](#_Toc9971)

[**4. References** 11](#_Toc5610)

# 1. Materials and methods

## 1.1 Collection and preprocessing of drug molecules related to α-syn

This study systematically retrieved small molecules targeting α-syn from the ChEMBL database [1], utilizing core keywords including "alpha-synuclein", "α-synuclein", "inhibitor", and "compound" to ensure documented target-specific activity. Following a meticulous curation of the dataset and the validation of molecular identifiers, chemical architectures, and bioactivity metrics, we excluded 540 compounds due to the absence of essential information, resulting in 10,421 candidate molecules. Subsequently, we employed structure-based deduplication utilizing the Tanimoto similarity coefficient with a cutoff of 0.8, which produced a final collection of 9,628 distinct small molecules. To ensure uniformity in potency evaluations, raw activity metrics (IC₅₀, EC₅₀, Ki, Kd, etc.) were transformed into pChEMBL values by standardizing units to molarity (M, e.g., nM×10⁻⁹) and employing the equation pChEMBL = –log₁₀ (activity_value (M)). Data points expressed as ranges or limits (e.g., ">10 μM", "<1 nM") were excluded to maintain consistency and precision for modeling purposes. Subsequently, the finalized dataset was randomly partitioned into training (N=7,701), validation (N=963), and independent test (N=964) subsets in an 8:1:1 ratio, facilitating subsequent machine learning model development and comprehensive performance evaluation.

## 1.2 Convolutional neural networks module

The initial convolutional layer utilizes a kernel size of 3 to concentrate on capturing local structural features from the ECFP representation. The following layers, the second and third, also employ 3-sized kernels but are designed to progressively increase the neurons’ receptive field through their stacked configuration. This architectural choice allows the model to effectively grasp longer-range structural relationships present in the fingerprint. Moreover, the operations performed by these later layers help reduce the dimensionality of the input features, thereby enhancing the model's efficiency and generalization ability. In each convolutional layer, the 1D fingerprint vector is convolved, followed directly by the application of a ReLU activation function, which introduces crucial nonlinear transformations to the feature extraction process. This process can be formally represented as:

$$\text{S}^{\text{(}\text{l}\text{+1)}}\text{=}\text{ReLU}\text{(}\text{CNN}\text{(}\text{W}^{\text{(}\text{l}\text{)}}\text{, }\text{b}^{\text{(}\text{l}\text{)}}\text{, }\text{S}^{\text{(}\text{l}\text{)}}\text{))}$$

where $\text{S}\text{∈}\text{R}^{\text{d}_{\text{ECFP}}\text{×}\text{d}_{\text{0}}}$refers to the feature representation of the ECFP, $\text{S}^{\text{(}\text{l}\text{)}}$denotes the hidden feature vector (or output vector) of layer$\text{l}$. $\text{S}^{\text{(0)}}$signifies the initial ECFP fingerprint vector (input vector), while $\text{W}^{\text{(}\text{l}\text{)}}$and $\text{b}^{\text{(}\text{l}\text{)}}$ represent the learnable weight matrix and bias vector for layer *l*, respectively.

## 1.3 The modified contextual graph attention network module (M-GAT)

Previous research has demonstrated that self-attention, cross-attention, and co-attention mechanisms can effectively capture inter-sequence dependencies to facilitate information fusion[2-5]. Inspired by contextual co-attention mechanisms, we propose a Modified Contextual Graph Attention Network (M-GAT). This architecture integrates the local feature extraction capability of GAT with the global feature modeling capacity of self-attention, enabling simultaneous capture of local information and enhancement of node-level interactions, thereby, increasing model sensitivity to critical features and consequently enhancing drug molecule prediction accuracy.

In the attention mechanism segment of this architecture, the contextual self-attention layer initially performs three distinct linear transformations on the input feature representation X, generating the classic Query (Q), Key (K), and Value (V)

$$\text{Q}\text{=}\text{W}_{\text{q}}\text{X}\text{, }\text{K}\text{=}\text{W}_{\text{k}}\text{X}\text{, }\text{V}\text{=}\text{W}_{\text{v}}\text{X}$$

where *W_q_, W_k_, W_v_* are learnable weight parameters.

Subsequently, 1D-CNNs are employed on the Key representation. Two separate 1D convolutional layers, utilizing kernel sizes of 3 and 5, respectively, facilitate feature extraction via convolution operations along the sequence dimension, yielding in two unique feature representations:

$\text{K}_{\text{CN}\text{N}_{\text{1×3}}}\text{=}\text{CN}\text{N}_{\text{1×3}}\text{(}\text{K}\text{), }\text{K}_{\text{CN}\text{N}_{\text{1×5}}}\text{=}\text{CN}\text{N}_{\text{1×5}}\text{(}\text{K}\text{)}$.

The $\text{K}_{\text{CN}\text{N}_{\text{1×3}}}$and $\text{K}_{\text{CN}\text{N}_{\text{1×5}}}$undergo concatenation to form a unified representation. This resultant vector is then processed through a linear transformation to revert to its original dimensionality. The outcome of this operation is the contextually augmented key representation, denoted as $\text{K}_{\text{ctx}}$

$\text{K}_{\text{ctx}}\text{=}\text{W}_{\text{K}}\text{concat}\text{(}\text{K}\text{, }\text{K}_{\text{CN}\text{N}_{\text{1×3}}}\text{, }\text{K}_{\text{CN}\text{N}_{\text{1×5}}}\text{)}$.

The attention weights are computed using Q and $\text{K}_{\text{ctx}}$

$$\text{attention}\text{\_}\text{scores}\text{=}\frac{\text{Q}\text{K}_{\text{ctx}}}{\sqrt{\text{d}_{\text{k}}}}$$

$$\text{attention}\text{\_}\text{weig}\text{h}\text{ts}\text{=}\text{softmax}\text{(}\text{attention}\text{\_}\text{scores}\text{)}$$

$$\text{F}_{\text{ctx}}\text{=}\text{attention}\text{\_}\text{weig}\text{h}\text{ts}\text{⋅}\text{V}$$

To better preserve the original information, a residual connection is incorporated by adding V directly back to the attention output *Attention_ctx_*

$\text{F}_{\text{final}}\text{=}\text{F}_{\text{ctx}}\text{+}\text{V}$.

## 1.4 Graph sample and aggregate module (GraphSAGE)

The GraphSAGE framework employs feature extraction techniques to capture both the topological and chemical attributes of molecular graphs, where nodes symbolize atoms in pharmaceutical compounds and edges represent covalent bonds. This research implements a two-layer architecture. In the initial layer, localized atom-level characteristics, such as the hybridization states of functional groups, are aggregated through neighborhood sampling. Conversely, the second layer focuses on gathering topological features, including the configurations of ring systems, through cross-structural aggregation. This architectural design facilitates the effective collection of multi-scale representations that integrate chemical features with topological properties in drug molecules. The following section details the specific process of feature transformation.

The feature propagation and update process at each layer can be formulated as follows:

$$\text{S}^{\text{(}\text{l}\text{+1)}}\text{=}\text{ReLU}\left( \text{SAGE}\left( \text{W}^{\left( \text{l} \right)}\text{, }\text{S}^{\left( \text{l} \right)}\text{, }\text{A} \right) \right)\text{, }\text{l}\text{=0, 1, 2}\text{}$$

where $\text{S}\text{∈}\text{R}^{\text{N}\text{×}\text{d}_{\text{0}}}$ refers to the feature matrix, A is the adjacency matrix of the molecule describing the connections between the various atoms, while $\text{W}^{\text{(}\text{l}\text{)}}$ represents the learnable weight matrix.

To obtain the final molecular representation, a global pooling layer is applied over the node features:

$$h_{\text{G}}\text{=}\text{Pooling}\text{(}\text{S}^{\text{(}\text{l}\text{)}}\text{)}$$

where $h_{\text{G}}$ denotes the graph-level representation, and $\text{Pooling}\text{(}\text{∙)}$ can be any permutation-invariant function such as mean, sum, or max pooling.

## 1.5 Training normalization and evaluation denormalization

To enhance convergence speed and prediction stability, we implement standardization (normalization) of target values (pChEMBL) across all samples. Specifically, the Standard Scaler[6, 7] method transforms target values in the training and validation sets according to:

$$\text{y}_{\text{norm}}\text{=}\frac{\text{y}\text{-}\text{μ}_{\text{train}}}{\text{σ}_{\text{train}}}$$

where *y* denotes the raw target value, while $\text{μ}_{\text{train}}$ and $\text{σ}_{\text{train}}$ represent the arithmetic mean and standard deviation of target values in the training set, respectively. This preprocessing strategy primarily mitigates scale discrepancies among annotated samples. Consequently, during model training, the network directly fits the normalized targets $\text{y}_{\text{norm}}$.

However, to preserve the interpretability and cross-model comparability of evaluation metrics (e.g., Mean Squared Error), we execute an inverse transformation during the testing and result-persisting phase. The denormalization operation follows:

$${\hat{\text{y}}}_{\text{orig}}\text{=}{\hat{\text{y}}}_{\text{norm}}\text{∙}\text{σ}_{\text{train}}\text{+}\text{μ}_{\text{train}}$$

where, ${\hat{\text{y}}}_{\text{norm}}\text{ }$signifies the model's normalized prediction output, whereas ${\hat{\text{y}}}_{\text{orig}}$ denotes the final prediction restored to the original physicochemical scale. Critically, it is upon this restored scale ${\hat{\text{y}}}_{\text{orig}}$ that we compute evaluation metrics such as MSE during testing.

## 1.6 Evaluation metrics

To quantitatively assess model performance, we employ three complementary metrics, including Mean Squared Error (MSE), Mean Absolute Error (MAE), and Pearson Correlation Coefficient, whose formulas are given as follows:

$$\text{MSE}\text{=}\frac{\text{1}}{\text{n}}\sum_{\text{i}\text{=1}}^{\text{n}} \text{(}\text{y}_{\text{i}}\text{-}{\hat{\text{y}}}_{\text{i}}\text{)}^{\text{2}}$$

$$\text{MAE}\text{=}\frac{\text{1}}{\text{n}}\sum_{\text{i}\text{=1}}^{\text{n}} \left| \text{y}_{\text{i}}\text{-}{\hat{\text{y}}}_{\text{i}} \right|$$

$$\text{r}\text{=}\frac{\sum_{\text{i}\text{=1}}^{\text{n}} \text{(}\text{y}_{\text{i}}\text{-}\bar{\text{y}}\text{)(}{\hat{\text{y}}}_{\text{i}}\text{-}\bar{\hat{\text{y}}}\text{)}}{\sqrt{\sum_{\text{i}\text{=1}}^{\text{n}} \text{(}\text{y}_{\text{i}}\text{-}\bar{\text{y}}\text{)}^{\text{2}}\sqrt{\sum_{\text{i}\text{=1}}^{\text{n}} \text{(}{\hat{\text{y}}}_{\text{i}}\text{-}\bar{\hat{\text{y}}}\text{)}^{\text{2}}}}}$$

where, $\text{y}_{\text{i}}$ is the *i*-th actual value, ${\hat{\text{y}}}_{\text{i}}$ is the $\text{i}$-th predicted value, n is the total number of samples, $\bar{\text{y}}$ and $\bar{\hat{\text{y}}}$ denote variable means, and $\text{r}\text{∈[}\text{-1, 1]}$ indicates correlation magnitude/direction.

## 1.7 Experimental setup

The SynGraphNet model was developed using PyTorch version 2.8.18 to effectively analyze both molecular 2D structural information and ECFP fingerprints. This innovative hybrid framework consists of a graph neural network component that utilizes a modified GAT architecture (with an output dimension of 35) integrated with SAGE convolution layers, designed to extract topological features. The model incorporates global max pooling to create fixed-length graph embeddings. Simultaneously, a convolutional component processes the fingerprint data through a series of three 1D convolutional layers, configured with channel sizes of 32, 64, and 128, and utilizing a constant kernel size of 3, all activated by the ReLU function. The fusion of features and subsequent prediction is performed using a multilayer perceptron structured as follows: 1025 input nodes, followed by 512 hidden nodes, and culminating in 1 output node. The training process employed the Adam optimizer with a learning rate set at 0.005, utilizing a batch size of 128 over the course of 1000 epochs.

# 2. Supplementary tables

**Table S1.** Atom features.

| Name | Description | Dim |
| --- | --- | --- |
| Atom type | Heavy atom type [C, N, O, S, F, P, Cl, Br, I, others] | 10* |
| Degree | Number of covalent bonds [0, 1, 2, 3, 4, 5, 6] | 7* |
| Implicit valence | Implicit valence of the atom [0, 1, 2, 3, 4, 5, 6] | 7* |
| Hybridization | [sp, sp2, sp3, sp3d, sp3d2] | 5* |
| Aromatic | Whether the atom is part of an aromatic system | 1 |
| Hydrogens | Number of connected hydrogens [0, 1, 2, 3, 4] | 5 |

*One-hot representation

**Table S2.** Performance of SynGraphNet under different hyperparameter settings.

| $\lambda_{L_{2}}$ | $\lambda_{KL}$ | learning rate | Validation MSE | Test MSE | Test MAE | R |
| --- | --- | --- | --- | --- | --- | --- |
| 1E-5 | 0.001 | 0.001 | 0.1761 | 0.2059 | 0.3405 | 0.3782 |
| 1E-5 | 0.001 | 0.0005 | 0.1777 | 0.1833 | 0.3292 | 0.4672 |
| 1E-5 | 0.0001 | 0.001 | 0.1783 | 0.1813 | 0.3281 | 0.4808 |
| 1E-5 | 0.0001 | 0.0005 | 0.1782 | 0.1832 | 0.3264 | 0.4717 |
| 1E-5 | 0.01 | 0.001 | 0.1821 | 0.1837 | 0.3334 | 0.4686 |
| 1E-5 | 0.01 | 0.0005 | 0.1774 | 0.1869 | 0.3298 | 0.4523 |
| 1E-4 | 0.001 | 0.001 | 0.1764 | 0.1812 | 0.3295 | 0.4776 |
| 1E-4 | 0.001 | 0.0005 | 0.1846 | 0.1940 | 0.3369 | 0.4162 |
| 1E-4 | 0.0001 | 0.001 | 0.1793 | 0.1816 | 0.3263 | 0.4741 |
| 1E-4 | 0.0001 | 0.0005 | 0.1796 | 0.1889 | 0.3336 | 0.4422 |
| 1E-4 | 0.01 | 0.001 | 0.1789 | 0.1831 | 0.3296 | 0.4691 |
| 1E-4 | 0.01 | 0.0005 | 0.1776 | 0.1869 | 0.3298 | 0.4523 |

**Table S3.** Comparative results with various machine learning methods.

|  | MSE | MAE | RMSE | R | R^2^ |
| --- | --- | --- | --- | --- | --- |
| SynGraphNet | 0.1812 | 0.3295 | 0.4257 | 0.4776 | 0.2460 |
| CatBoost | 0.1848 | 0.3271 | 0.4296 | 0.4649 | 0.2115 |
| RF | 0.1849 | 0.3277 | 0.4299 | 0.4647 | 0.2106 |
| LightGBM | 0.1853 | 0.3285 | 0.4305 | 0.4610 | 0.2083 |
| XGBoost | 0.1856 | 0.3299 | 0.4309 | 0.4669 | 0.2068 |
| SVR | 0.1862 | 0.3328 | 0.4315 | 0.4626 | 0.2044 |
| Gradient Boosting | 0.1885 | 0.3349 | 0.4342 | 0.4468 | 0.1945 |
| KNN | 0.2010 | 0.3377 | 0.4484 | 0.4408 | 0.1411 |
| Extra Tree | 0.2031 | 0.3345 | 0.4507 | 0.4337 | 0.1321 |
| Bayesian | 0.2032 | 0.3443 | 0.4508 | 0.3737 | 0.1317 |
| Lars | 0.2036 | 0.3449 | 0.4513 | 0.3727 | 0.1300 |
| Bagging | 0.2056 | 0.3381 | 0.4535 | 0.3987 | 0.1215 |
| Orthogonal Matching Pursuit | 0.2091 | 0.3478 | 0.4573 | 0.3545 | 0.1065 |
| ARD Regression | 0.2109 | 0.3522 | 0.4592 | 0.3423 | 0.0990 |
| Ridge | 0.2187 | 0.3572 | 0.4677 | 0.3450 | 0.0655 |
| AdaBoost | 0.2235 | 0.3866 | 0.4728 | 0.3470 | 0.0449 |
| Huber | 0.2248 | 0.3507 | 0.4741 | 0.3261 | 0.0395 |
| Lasso Lars | 0.2342 | 0.3619 | 0.4840 | Nan | -0.0007 |
| Lasso | 0.2342 | 0.3619 | 0.4840 | Nan | -0.0007 |
| Elastic Net | 0.2342 | 0.3619 | 0.4840 | Nan | -0.0007 |
| Decision Tree | 0.3229 | 0.3963 | 0.5682 | 0.2905 | -0.3794 |
| Passive Aggressive | 0.3538 | 0.4663 | 0.5948 | 0.2108 | -0.5118 |
| MLP | 0.4543 | 0.5137 | 0.6740 | 0.2255 | -0.9409 |
| Kernel Ridge | 24.4440 | 4.9920 | 4.9441 | 0.3450 | -103.4343 |
| Gaussian Process | 27.6670 | 4.8335 | 5.2609 | 0.0489 | -117.2467 |
| TheilSen | 2.13E+05 | 49.5241 | 461.0422 | -0.0049 | -9.08E+5 |
| RANSAC | 3.94E+16 | 8.20E+6 | 1.98E+8 | 0.0112 | -1.68E+17 |
| Linear Regression | 1.87E+17 | 1.39E+7 | 4.3223E+8 | 0.0112 | -7.98E+17 |

**Table S4.** Systematic evaluation of model performance across combinations of Morgan Fingerprint parameters and regularization hyperparameters.

|  | Radius | ${[\lambda}_{L_{2}}{,\lambda}_{KL},$learning rate] | Validation MSE | Test MSE | Test MAE | R |
| --- | --- | --- | --- | --- | --- | --- |
| Morgan-1024 | 1 | [1E-4, 0.001, 0.0005] | 0.1762 | 0.1860 | 0.3295 | 0.4561 |
|  | 1 | [1E-4, 0.0001, 0.001] | 0.1803 | 0.1830 | 0.3297 | 0.4725 |
|  | 1 | [1E-5, 0.0001, 0.001] | 0.1787 | 0.1835 | 0.3290 | 0.4684 |
| Morgan-1024 | 3 | [1E-4, 0.001, 0.0005] | 0.1811 | 0.1858 | 0.3310 | 0.4626 |
|  | 3 | [1E-4, 0.0001, 0.001] | 0.1796 | 0.1877 | 0.3386 | 0.4497 |
|  | 3 | [1E-5, 0.0001, 0.0005] | 0.1808 | 0.1839 | 0.3328 | 0.4633 |
| Morgan-2048 | 1 | [1E-4, 0.001, 0.0005] | 0.1821 | 0.1835 | 0.3317 | 0.4671 |
|  | 1 | [1E-4, 0.0001, 0.001] | 0.1814 | 0.1861 | 0.3278 | 0.4661 |
|  | 1 | [1E-5, 0.001, 0.0005] | 0.1790 | 0.1828 | 0.3299 | 0.4692 |
| Morgan-2048 | 3 | [1E-4, 0.001, 0.0005] | 0.1770 | 0.1897 | 0.3317 | 0.4415 |
|  | 3 | [1E-5, 0.0001, 0.0005] | 0.1759 | 0.1851 | 0.3362 | 0.4659 |
|  | 3 | [1E-5, 0.001, 0.0005] | 0.1760 | 0.1877 | 0.3361 | 0.4570 |

# Supplementary figures


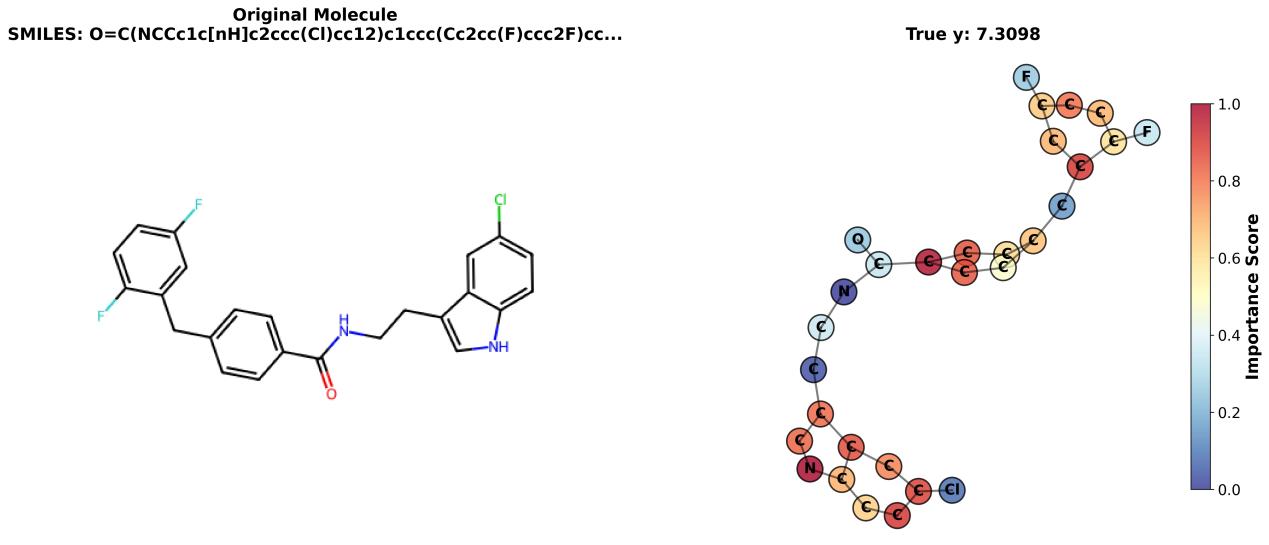


**Figure S1. Molecular structure and Atom-Wise importance visualization of CHEMBL3898223.** The left panel shows the molecular structure of the compound together with its SMILES representation. The right panel illustrates the atom-wise importance distribution calculated by the model, where the color gradient from blue to red indicates increasing importance. The true property value of the compound is **7.3098.**


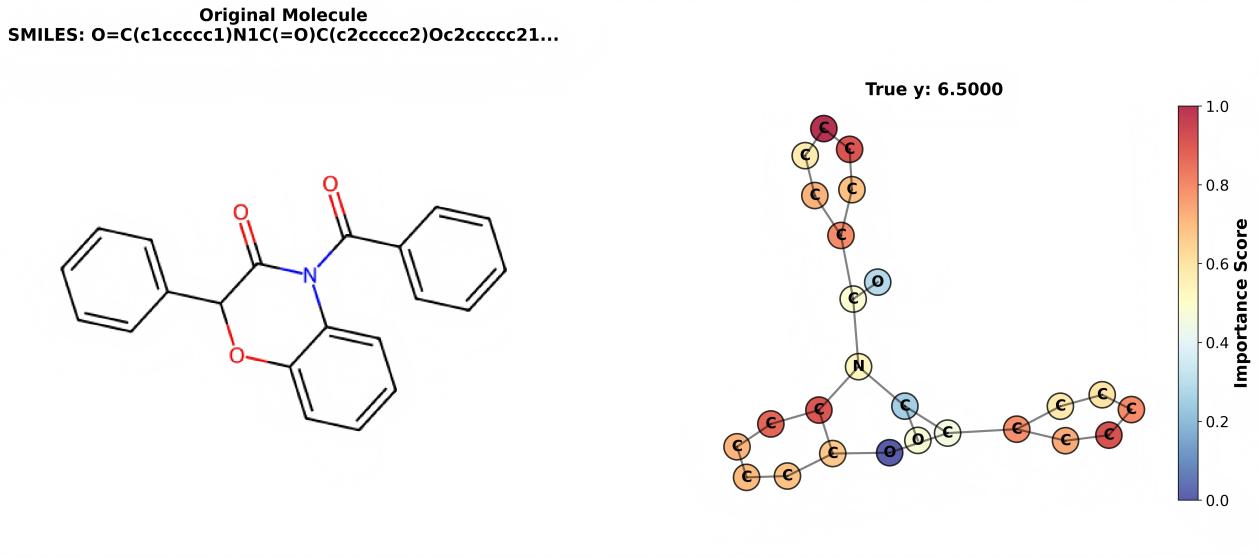


**Figure S2. Molecular structure and Atom-Wise importance visualization of CHEMBL1487074.** The left panel presents the molecular structure of the compound along with its SMILES representation. The right panel shows the atom-wise importance distribution computed by the model, where the color gradient from blue to red indicates increasing atomic importance. The true property value of the compound is **6.5000.**

**
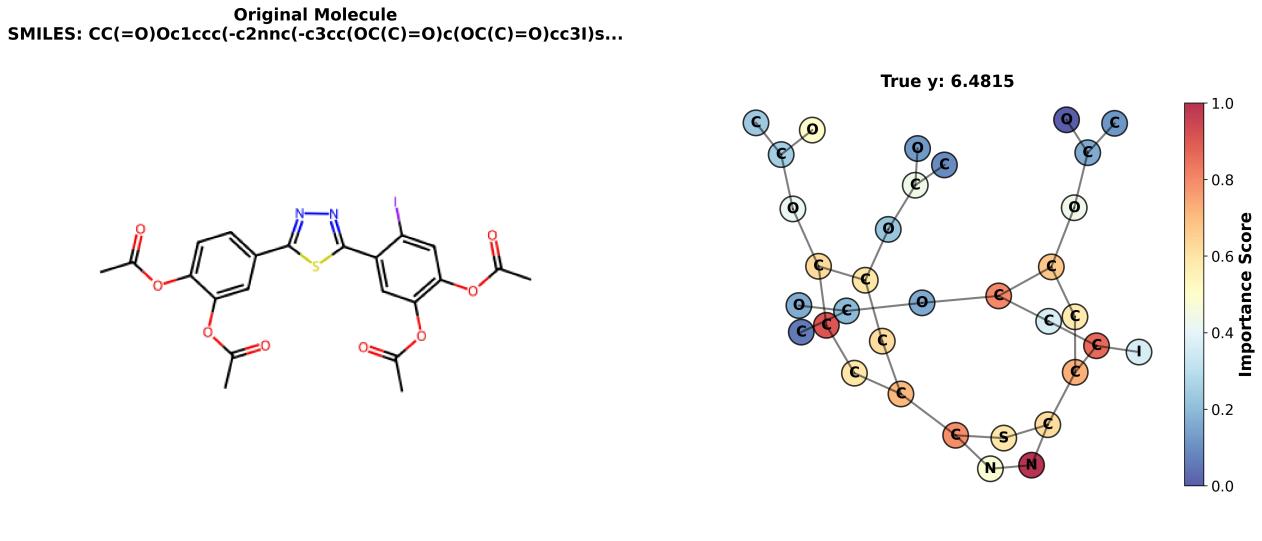
**


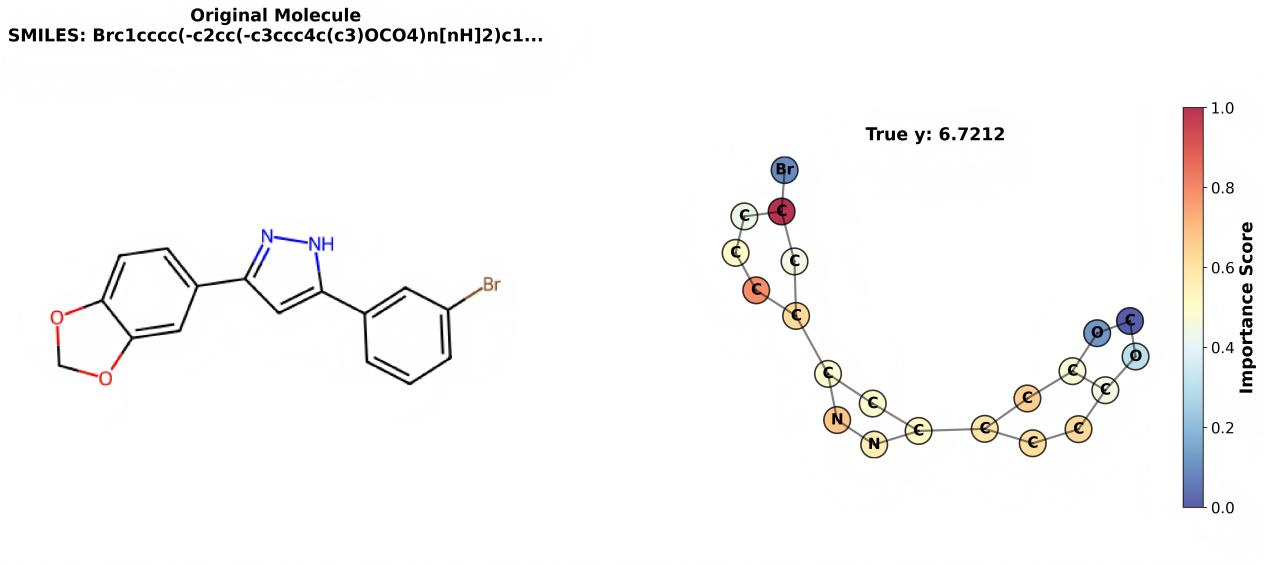
**Figure S3. Molecular structure and Atom-Wise importance visualization of CHEMBL3663959.** The left panel displays the molecular structure of the compound together with its SMILES representation. The right panel illustrates the atom-wise importance distribution computed by the model, where the color gradient from blue to red indicates increasing importance. The true property value of the compound is **6.4815.**

**Figure S4. Molecular structure and Atom-Wise importance visualization of CHEMBL4748063.**The left panel presents the molecular structure of the compound along with its SMILES representation. The right panel shows the atom-wise importance distribution calculated by the model, where the color gradient from blue to red indicates increasing atomic importance. The true property value of the compound is **6.7212**.


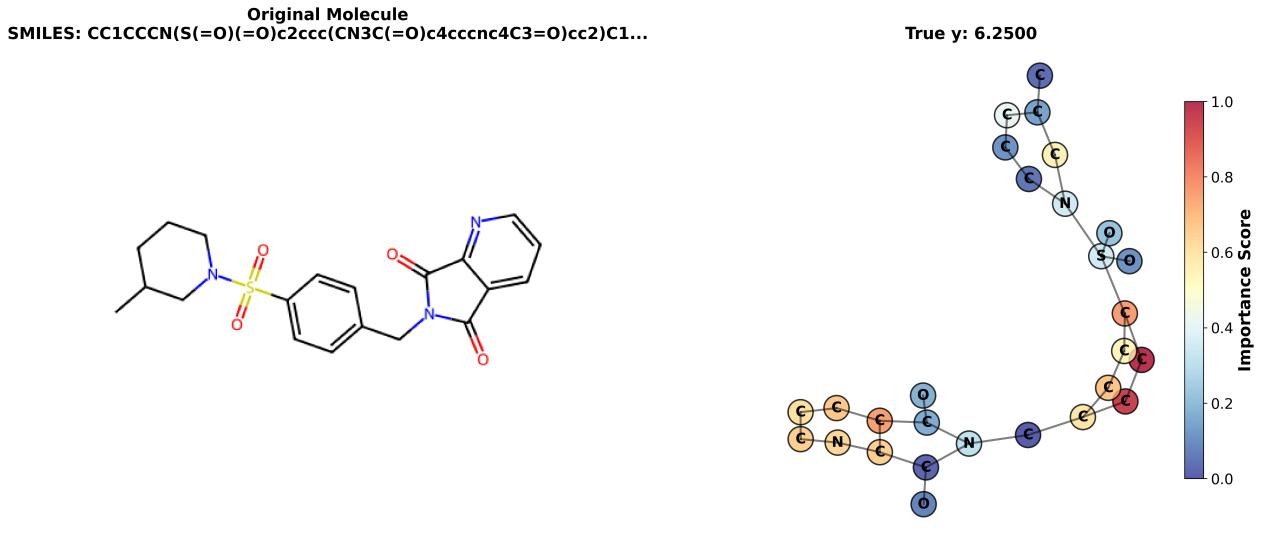


**Figure S5. Molecular sStructure and Atom-Wise importance visualization of CHEMBL1463430.** The left panel shows the molecular structure of the compound together with its SMILES representation. The right panel depicts the atom-wise importance distribution computed by the model, where the color gradient from blue to red indicates increasing atomic importance. The true property value of the compound is **6.2500**.


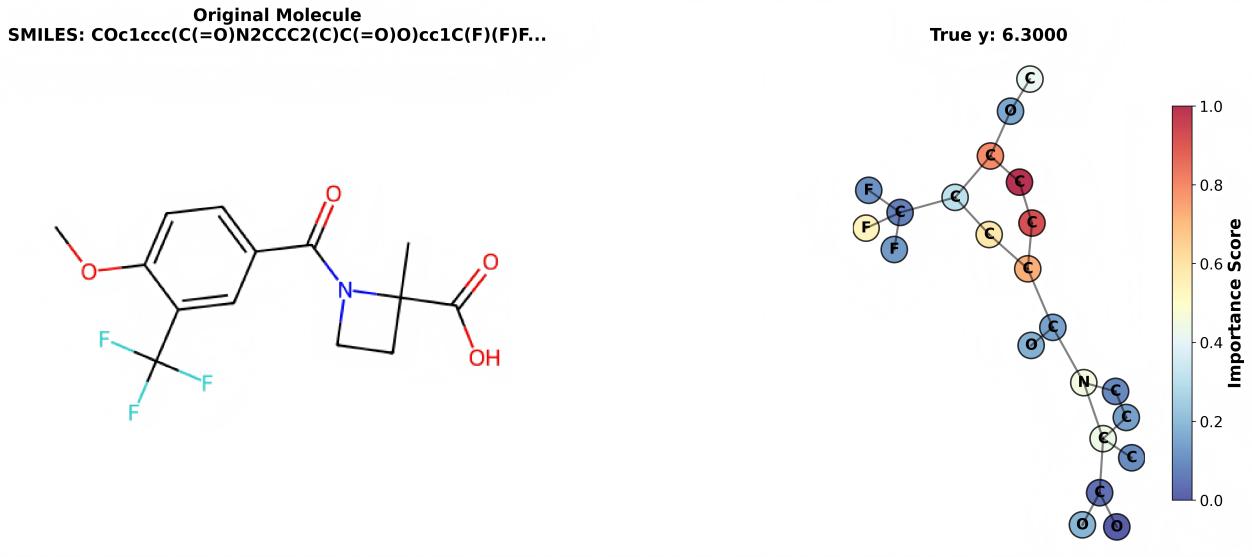


**Figure S6. Molecular structure and Atom-Wise importance visualization of CHEMBL2358775.** The left panel presents the molecular structure of the compound along with its SMILES representation. The right panel displays the atom-wise importance distribution computed by the model, where the color gradient from blue to red indicates increasing atomic importance. The true property value of the compound is **6.3000**.

# References

[1] R. Manguinhas, P.A. Serra, R.B. Soares, R. Rosell, N. Gil, N.G. Oliveira, R.C. Guedes, Unveiling Novel ERCC1-XPF Complex Inhibitors: Bridging the Gap from In Silico Exploration to Experimental Design, Int J Mol Sci 25(2) (2024).

[2] N.Q. Nguyen, G. Jang, H. Kim, J. Kang, Perceiver CPI: a nested cross-attention network for compound-protein interaction prediction, Bioinformatics 39(1) (2023).

[3] Y. Li, Y. Wei, S. Xu, Q. Tan, L. Zong, J. Wang, Y. Wang, J. Chen, L. Hong, Y. Li, AcrNET: predicting anti-CRISPR with deep learning, Bioinformatics 39(5) (2023).

[4] P.A. Campana, Z. Nikoloski, Self- and cross-attention accurately predicts metabolite-protein interactions, NAR Genom Bioinform 5(1) (2023) lqad008.

[5] Y. Fang, F. Xu, L. Wei, Y. Jiang, J. Chen, L. Wei, D.Q. Wei, AFP-MFL: accurate identification of antifungal peptides using multi-view feature learning, Brief Bioinform 24(1) (2023).

[6] W. Ahmed, M.A. Wani, P. Plawiak, S. Meshoul, A. Mahmoud, M. Hammad, Machine learning-based academic performance prediction with explainability for enhanced decision-making in educational institutions, Sci Rep 15(1) (2025) 26879.

[7] J. Ren, R. Loughnan, B. Xu, W.K. Thompson, C.C. Fan, Estimating the total variance explained by whole-brain imaging for zero-inflated outcomes, Commun Biol 7(1) (2024) 836.
